# Expression of the B-Cell Receptor Component CD79a on Immature Myeloid Cells Contributes to Their Tumor Promoting Effects

**DOI:** 10.1371/journal.pone.0076115

**Published:** 2013-10-16

**Authors:** Dror Luger, Yu-an Yang, Asaf Raviv, Douglas Weinberg, Subhadra Banerjee, Min-Jung Lee, Jane Trepel, Li Yang, Lalage M. Wakefield

**Affiliations:** 1 Laboratory of Cancer Biology and Genetics, National Cancer Institute, Bethesda, Maryland, United States of America; 2 Medical Oncology Branch, Center for Cancer Research, National Cancer Institute, Bethesda, Maryland, United States of America; Southern Illinois University School of Medicine, United States of America

## Abstract

The role of myeloid derived suppressor cells (MDSCs) in promoting tumorigenesis is well-established, and significant effort is being made to further characterize surface markers on MDSCs both for better diagnosis and as potential targets for therapy. Here we show that the B cell receptor adaptor molecule CD79a is unexpectedly expressed on immature bone marrow myeloid cells, and is upregulated on MDSCs generated in multiple different mouse models of metastatic but not non-metastatic cancer. CD79a on MDSCs is upregulated and activated in response to soluble factors secreted by tumor cells. Activation of CD79a on mouse MDSCs, by crosslinking with a specific antibody, maintained their immature phenotype (CD11b+Gr1+), enhanced their migration, increased their suppressive effect on T cell proliferation, and increased secretion of pro-tumorigenic cytokines such as IL-6 and CCL22. Furthermore, crosslinking CD79a on myeloid cells activated signaling through Syk, BLNK, ERK and STAT3 phosphorylation. In vivo, CD79+ myeloid cells showed enhanced ability to promote primary tumor growth and metastasis. Finally we demonstrate that CD79a is upregulated on circulating myeloid cells from lung cancer patients, and that CD79a+ myeloid cells infiltrate human breast tumors. We propose that CD79a plays a functional role in the tumor promoting effects of myeloid cells, and may represent a novel target for cancer therapy.

## Introduction

The existence of cancer-induced myeloid-derived suppressor cells (MDSCs) is well-established. Tumorigenesis is almost invariably associated with the expansion of an immature myeloid cell population that shows varying degrees of differentiation blockade and can be activated to an immune suppressive phenotype [1]. Patients with cancer can show up to a ten-fold increase in circulating MDSCs, and MDSCs accumulate in tumors, lymph nodes, and spleen, constituting as much as 40% of cells in the spleen in certain mouse models [1]. However the importance of these cells in supporting tumor growth and metastasis formation has only recently been appreciated [1]–[3]. MDSCs have been shown to be involved in a wide variety of tumor promoting mechanisms, including angiogenesis [4], [5], lymphangiogenesis [6], extracellular matrix remodeling [7], immune suppression [8], and formation of the pre-metastatic niche [7], [9]. The immunosuppressive effects of MDSCs are mediated by multiple mechanisms, including expression of T cell suppressive factors such as iNOS, Arginase-1, reactive oxygen species and peroxynitrite; polarization of macrophages towards an protumorigenic M2 phenotype; inhibition of dendritic cell and natural killer cell function; and induction and recruitment of regulatory T cells (T_reg_) [1]–[3] [10], [11]. Currently there is a strong interest in developing therapeutic strategies to block the expansion, mobilization and activities of this cell population. To achieve this goal, an intensive effort is needed to further characterize MDSC phenotypes and biology.

The common characteristics of MDSCs in almost all tumor types are their myeloid origin and immature phenotype. However MDSCs are phenotypically diverse, with many different subpopulations expressing different combinations of cell surface markers depending on the cancer type and stage [12], [13]. In mice the hallmark of MDSCs is the co-expression of CD11b+ and Gr1+, reflecting their immature status and close relationship to the immature myeloid cells that exist in the normal bone marrow (BM). However among cells with this common characteristic, several subpopulations have been identified that show different levels of Gr1expression (high/intermediate), as well as different proportions of the Gr1 components Ly6G and Ly6C. Granulocytic MDSCs are Ly6G^+^Ly6C^lo^ while monocytic MDSCs are Ly6G^−^Ly6C^+^, and although both subsets are immunosuppressive, they deploy different mechanisms [1]. In human cancer patients, characterization of MDSCs is more complicated since there is no human analog of the Gr1 (Ly6C/G) marker. Characterization of MDSCs in humans has included a larger number of cell surface markers (CD11b, CD33, CD14, CD15, CD34, CD13 and others), with one widely used marker combination being Lin1^−/low^/HLA-DR^−^/CD11b^+^/CD33^+^ [14], [15]). From the standpoint of therapeutic targeting, it will be important to identify markers that are differentially expressed between normal immature myeloid cells and MDSCs, as well as to determine whether any of the markers actually play a functional role in the tumor-promoting activities of the MDSCs.

CD79a (also known as Ig-α or mb-1) is an integral membrane protein that is highly conserved among many species [16]. It is expressed at the very early stages of B cell development [17], and expression of CD79a is maintained until the last stage of maturation before differentiation to plasma cells [18], [19]. In normal conditions, CD79a forms a disulfide-linked heterodimer with CD79b, and non-covalently assembles together with membrane bound IgM to form the B cell receptor signaling complex (BCR) [20], [21]. The role of the dimer CD79a/b is to transmit the signal generated by antigen binding to the BCR into the cell for induction of B cell activation. Following engagement of the BCR, CD79a and CD79b are phosphorylated on their intracellular immunoreceptor tyrosine-based activation motif (ITAM) domains by Src family kinases, leading to recruitment and activation of the kinase Syk. Syk activation then triggers a signal transduction cascade leading to cytoskeletal reorganization and changes in gene expression that affects B-cell fate. The ITAM motifs of both CD79a and CD79b were found to be essential for B cell maturation [22]. Additionally CD79a/b may induce the early stages of B-cell maturation and maintain B cell survival in the periphery by tonic antigen-independent signaling [21], [23].

The expression of CD79a was originally thought to be highly specific for the B-cell lineage, and in disease its presence in combination with blast antigens was considered diagnostic for B-cell acute lymphoblastic leukemia [24], [25]. However, CD79a has also been found in some cases of myeloid leukemia, termed biphenotypic leukemia, in which CD79a was coexpressed with myeloid markers on bone marrow blast cells [26], [27]. Currently it is not clear whether the expression of CD79a in these biphenotypic leukemias is just a marker of aberrant lineage commitment and differentiation in tumor cells, or whether it plays a functional role independent of the BCR. In the present study, while analyzing the role of B-cells in metastatic cancer progression, we made the unexpected finding that CD79a is expressed on naïve immature BM myeloid cells, and on MDSCs in tumor-bearing animals. We provide evidence that CD79a plays an important functional role in maintaining the immature, immune suppressive phenotype of MDSCs and in inducing the secretion of protumorigenic cytokines. Tumor-induced CD79a expression on MDSCs was also observed in human cancer patients, suggesting that CD79a on MDSCs may be a novel target for cancer therapy.

## Results

### Increasing metastatic efficiency is associated with increased myeloid cells and reduced B-cells in breast cancer models

**Figure 1:**
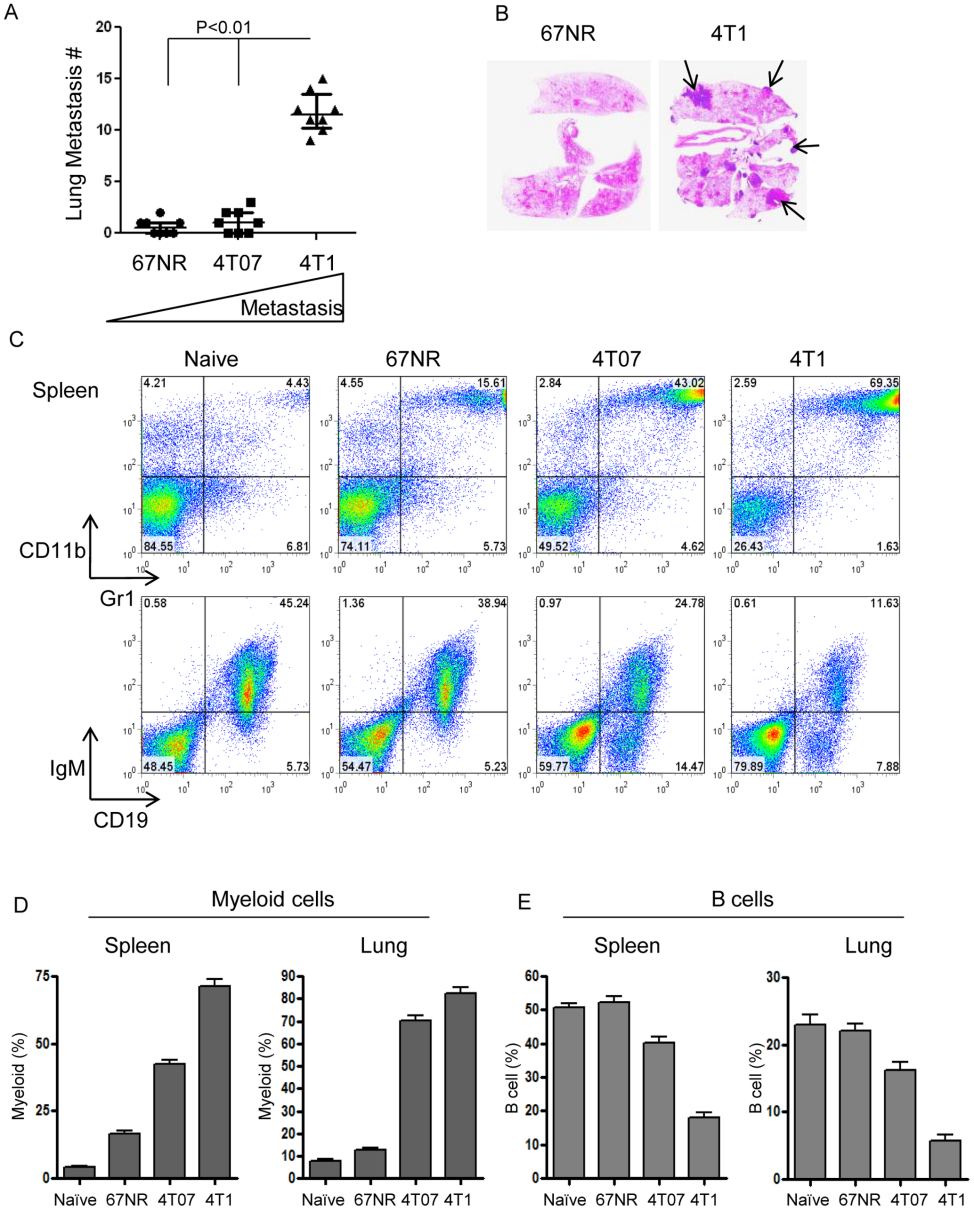
Increased myeloid cells and reduced B-cells associated with increasing metastatic efficiency in breast cancer models. Metastatic 4T1 and poorly- or non-metastatic 4T07 and 67NR cell lines were implanted orthotopically in Balb/c mice, and the extent of lung metastasis and the relative representation of immature myeloid (CD11b^+^Gr1^+^) and mature B (CD19^+^IgM^+^) cells in lungs and spleen were analyzed 28 days post-inoculation. (A) Number of histologically-detectable metastases in lungs from mice inoculated with the different cell lines. N = 8 mice/group. Bars show median and interquartile range. (B) Representative histological cross-sections of lungs from mice with 67NR or 4T1 tumors. Arrows indicate metastases. (C) Representation of immature myeloid cells (CD11b^+^Gr1^+^) and mature B cells (IgM^+^CD19^+^) in spleens from the different groups as assessed by flow cytometry. (D) Histograms summarizing relative numbers of immature myeloid cells and mature B-cells as a percent of viable leukocytes in naïve and tumor-bearing mice. N = 5 mice/group.

To screen the major immune responses in metastatic vs non metastatic breast cancer models we inoculated Balb/c mice orthotopically with a well-established panel of murine breast cancer cell lines with different metastatic abilities; the metastatic 4T1, the invasive but poorly metastatic 4T07 and the non-metastatic 67NR [28]. We confirmed that only the 4T1 cells generated significant numbers of metastatic colonies in the lungs (Figure 1A,B). 4T1 tumors have previously been shown to drive the generation of large numbers of MDSCs [29]. Here we showed that the increasing metastatic ability of the cell lines correlated with progressively increased levels of CD11b+Gr1+ MDSCs, both in the spleen, and in the lungs which are the principal target organ for metastasis when this model is implanted orthotopically (Figure 1C,D). The increase in MDSCs was accompanied by a significant reduction of cells in the B cell compartment (Figure 1C,E).

### The B cell receptor subunit CD79a is expressed on the MDSC population that is expanded by metastatic tumors

**Figure 2:**
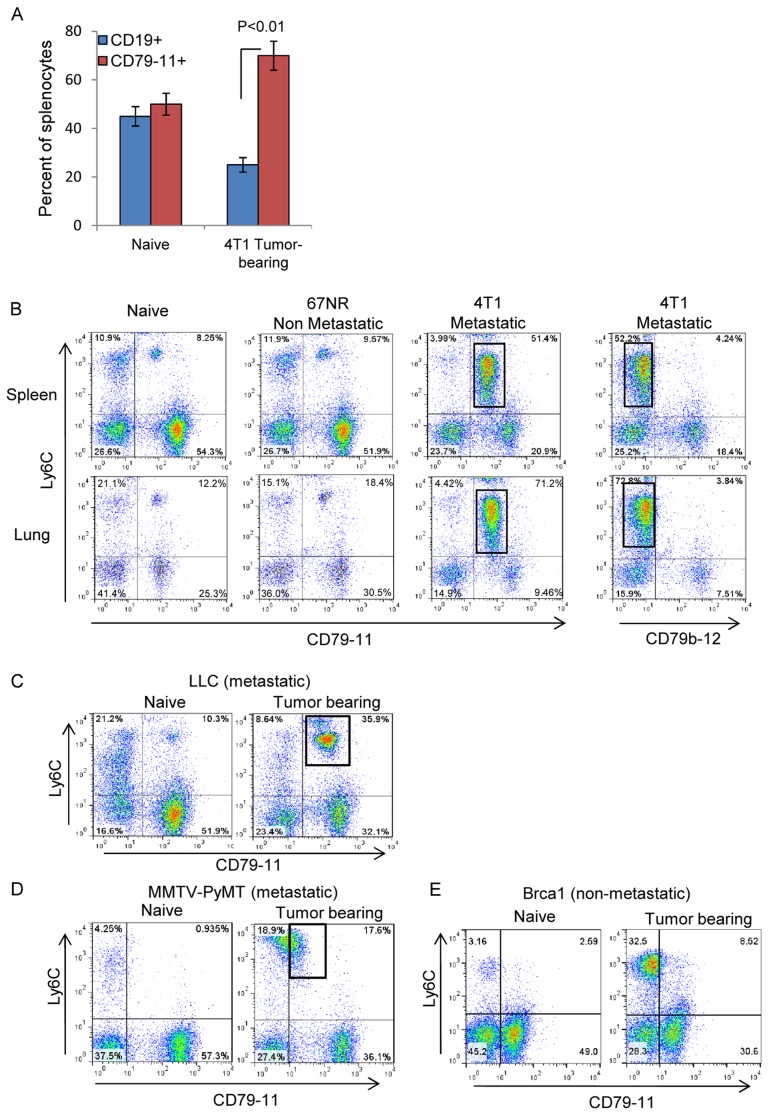
Expression of the B-cell receptor subunit CD79a on MDSCs induced by metastatic tumors. CD79a/b expression on the Ly6C^+^ myeloid cell population in multiple tumor models was assessed by flow cytometry. (A) Expression of the B-cell markers CD19 and CD79a/b on splenocytes from naïve and 67NR and 4T1 tumor-bearing mice. (B) Ly6C and CD79a/b expression on viable leukocytes from lungs and spleens of Balb/c mice bearing 67NR or 4T1 tumors at day 28 post-inoculation. The box indicates the immature myeloid population. CD79 on myeloid cells is recognized by the CD79-11 but not the CD79-12 antibody. (C) CD79a expression on MDSCs was also evaluated in the Lewis Lung Carcinoma transplantable metastatic lung cancer model (C57Bl/6 background). (D,E) Similar evaluation was done in two genetically-engineered models of breast cancer; the metastatic MMTV-PyMT model (D) on the FVB/N background (age 3 months) and the non-metastatic Brca1 model (E) on a mixed genetic background (age 6 months).

Since not much is known about the involvement of B cells in the development of solid tumors, we analyzed the B cells further using multiple markers, including the B cell receptor-associated molecules CD79b and CD79a. To evaluate the expression of these components we used several different antibodies. The clone HM79-12 (termed CD79-12) is specific to the extracellular domain of CD79b. There is no good monoclonal antibody specific for the extracellular domain of mouse CD79a, so we used the monoclonal antibody clone HM79-11 (termed CD79-11), described as specific for the complex CD79a/b [30]. While there was a decrease in CD19^+^ splenocytes in 4T1 tumor-bearing mice compared with naïve mice or mice bearing non-metastatic 67NR tumors, we observed an unexpected increase in splenocytes that were positive for CD79-11 in 4T1 tumor-bearing mice (Figure 2A). We therefore investigated further to determine what cell type was staining for CD79-11 in the tumor-bearing mice. Ly6C was used as a single marker for bone marrow myeloid cells and MDSCs since we showed that CD11b, Gr1 and Ly6+ were all coexpressed at high levels in these cells (Supplemental Figure S1). We found that the majority of Ly6C^+^ MDSCs generated by the metastatic 4T1 model were positive for CD79-11 staining (Figure 2B). In contrast, a much smaller fraction of Ly6C^+^ cells showed CD79-11 positivity in naïve mice, or mice bearing the non-metastatic 67NR tumors (Figure 2B). Since the CD79b-specific CD79-12 antibody did not detect this subpopulation of myeloid cells (Figure 2B), we concluded that the bi-specific CD79-11 antibody was recognizing CD79a on the myeloid cells. We then examined other metastatic models in different mouse strains. In the transplantable metastatic Lewis Lung carcinoma (LLC) model which is syngeneic to C57Bl/6, peripheral myeloid cells expanded by the tumor expressed CD79a (detected by CD79-11) (Figure 2C). We observed similar myeloid cells expressing CD79a in the genetically engineered MMTV-PyMT metastatic mammary cancer model on the FBV/N mouse strain (Figure 2D). In contrast, in the genetically engineered non-metastatic BrCa1 mammary cancer model (on a mixed mouse strain), the expanded myeloid cells did not upregulate CD79a (CD79-11) (Figure 2E).

### CD79a is expressed on immature myeloid cells in naïve mice

While evaluating CD79a expression in tumor-bearing mice, we also observed a small myeloid cell population that expresses CD79a in the spleen and lungs of naïve mice. Thus we hypothesized that CD79a is a member of the constellation of cell-surface markers expressed on immature myeloid cells. We show here for the first time that CD79a is expressed on the majority of naïve myeloid BM progenitors, as well as on a smaller but significant proportion of the myeloid cell population in the blood, lungs and spleen (Supplementary Figure S2A). Unlike mature B cells which express both CD79a and CD79b, the immature myeloid cells expressed solely CD79a (Supplementary Figure S2B). We further show that these immature myeloid cells do not express other B cell markers, except for a small population that is separated from the main immature myeloid cell population and may represent plasmacytoid dendritic cells (Supplementary Figure S2C).

**Figure 3:**
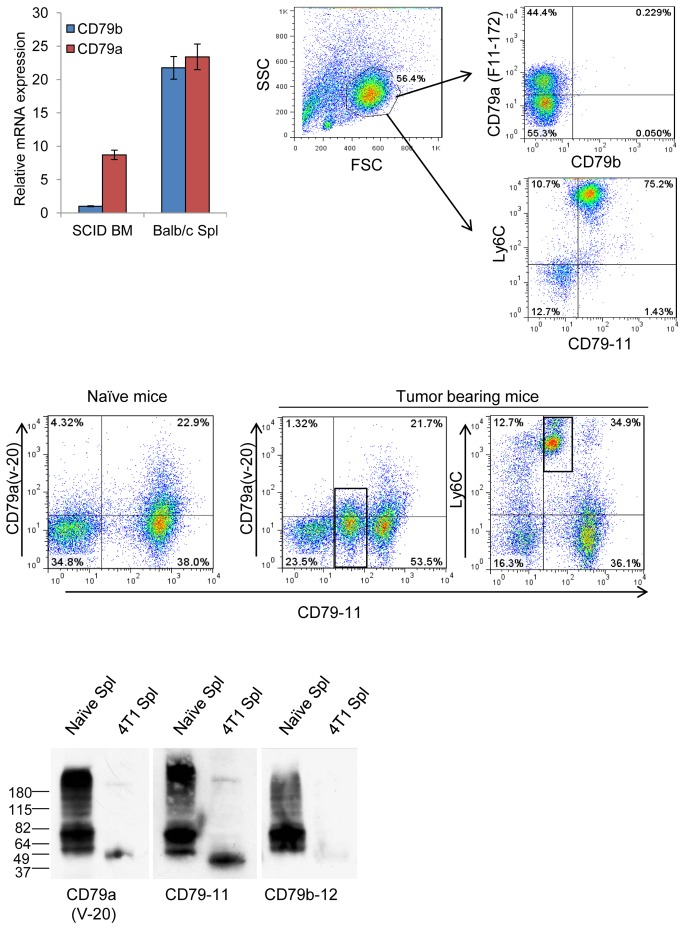
Expression of CD79a mRNA and protein in bone marrow cells from B-cell deficient mice. (A) RNA was extracted from BM cells of naïve SCID mice and from spleen of naïve Balb/c mice and CD79a and CD79b mRNA expression were measured by qRT-PCR. RNA levels were normalized to PPIAand presented relative to the level of CD79b mRNA in SCID BM. Results are mean +/− SEM; n = 3. (B) BM-derived leukocytes from naïve SCID mice were analyzed by flow cytometry for intracellular epitopes of CD79a using the anti-mouse CD79a clone F11-172. (C) To assess the extracellular expression of CD79a, BM cells from mice bearing LLC tumors were co-stained with anti CD79-11 together with the polyclonal anti-CD79a(v-20), which showed a weaker pattern of staining than CD79-11. Boxes indicate the myeloid cells. (D) CD79a/b protein in splenocytes from naïve and 4T1 tumor-bearing mice was assessed by western blot under reducing conditions. Data shown are representative of three independent experiments.

B cells and myeloid cells share the expression of certain major transcription factors such as PU.1 [31], and both cell types have the plasticity to gain characteristics of the other lineage under pathological conditions [32]. In particular, B cells have the ability to gain myeloid markers in the presence of a combination of growth factors (mainly of the colony stimulating factors (CSF)) [33]. In order to examine whether the myeloid cells expressing CD79a were of B cell origin, we used the SCID mouse strain that lacks lymphocytes. We demonstrated CD79a expression in BM myeloid cells from SCID mice both by RT-PCR (Figure 3A) and by FACS staining with the anti-CD79-11 antibody and an additional anti-CD79a (F11-172) antibody that recognizes the intracellular domain of CD79a (Figure 3B). Thus the myeloid cells expressing CD79a are not of B-cell origin. We then further confirmed expression of CD79a on myeloid cells in immunocompetent mice using a polyclonal antibody CD79a(v-20) (Santa Cruz) that specifically recognizes the extracellular domain of CD79a. Staining with CD79a(v-20) together with CD79-11 showed that both antibodies recognize B cells from naïve spleens as well as myeloid cells from spleens of tumor bearing mice (Figure 3C). However, CD79a(v-20) showed only partial positivity in both B-cell and myeloid cell compartments, suggesting that CD79-11 and CD79(v-20) differ in their efficiency for detecting CD79a. To test whether CD79a expressed on myeloid cells is structurally different from that expressed on B cells, protein was extracted for western blot analysis from splenocytes of naive mice or from mice with a heavy 4T1 tumor burden such that nearly 90% of the splenocytes were immature myeloid cells. In the normal B cells, CD79a appeared as multiple bands whereas CD79a in myeloid cells was a single band at a lower MW (Figure 3D), suggesting that the CD79a in the myeloid cells may represent a shorter splice variant or a different glycoform.

### Factors secreted by metastatic tumor cells expand CD79a-expressing BM myeloid cells and enhance their migration

**Figure 4:**
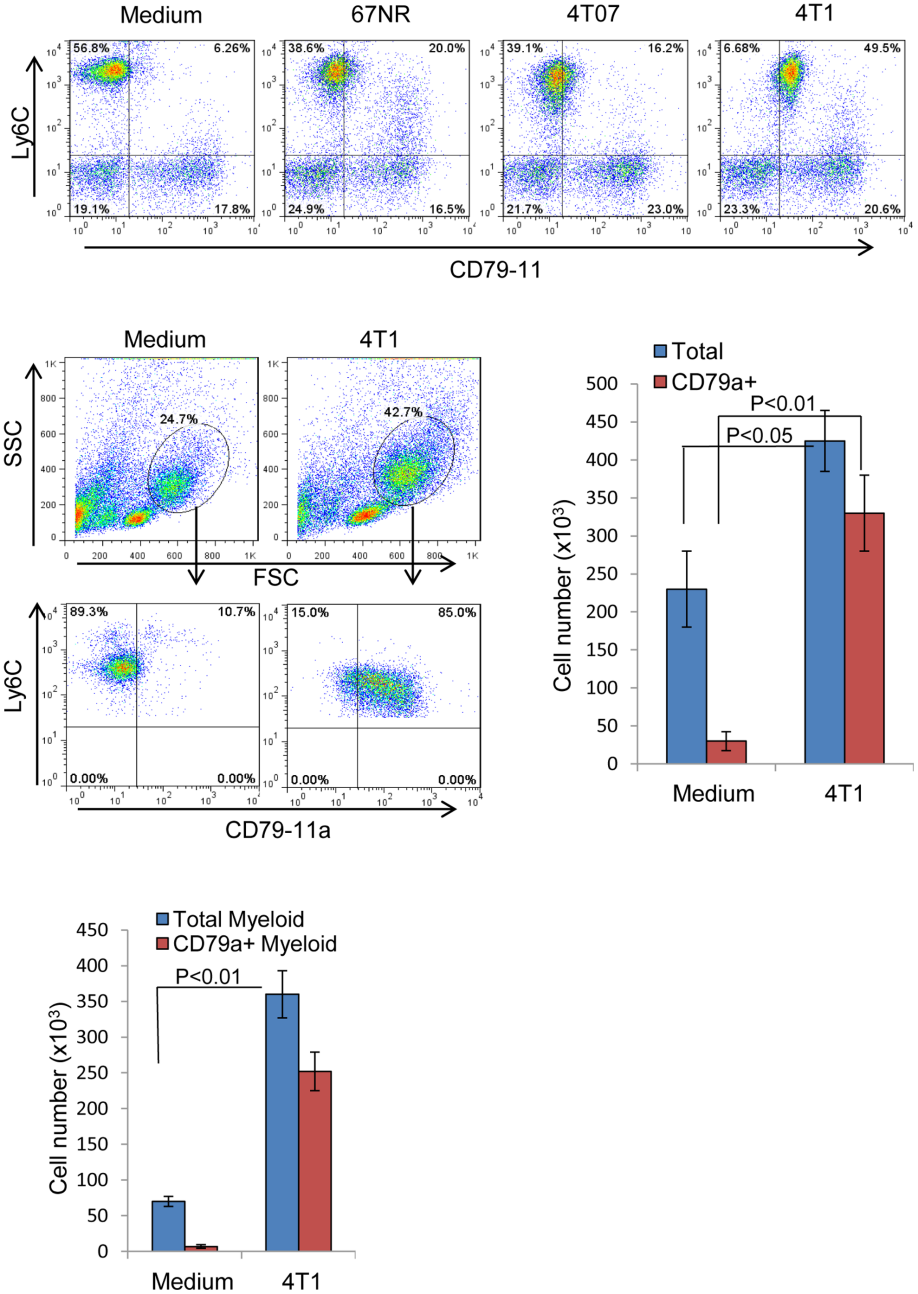
Tumor cell-secreted factors upregulate CD79a on myeloid cells and induce their expansion and migration. (A) The ability of tumor cells of varying metastatic potential to induce expression of CD79a on immature myeloid cells from bone marrow of naïve mice was assessed using a Transwell® system (0.4 µm pore size) that prevented contact between tumor and bone marrow-derived cells. BM cells were co-incubated with tumor cells of medium alone for 48 h and then BM cells were analyzed by flow cytometry. (B) Representative FACS plots and histograms showing the effect of 4T1 secreted factors on the expansion of BM myeloid cells expressing CD79a, tested using a Transwell® system as in A. (C) Migration of BM cells in response to 4T1 secreted factors was tested in a Transwell® system as above, but using a 3.0 µm pore-size membrane to allow migration of BM cells towards the 4T1 cells in the well below. Cells that migrated through the membrane into the well below were collected, counted and analyzed by FACS for CD79a expression. Results are mean +/- SEM; n = 4 determinations.

Based on the significant effect of primary tumors from metastatic models on the expansion of CD79a+ immature myeloid cells, we hypothesized that soluble factors secreted by these tumors might mediate this effect (differential cytokine secretion by the metastatic 4T1and the non-metastatic 67NR cell lines is described in supplementary table s1). To test this hypothesis we used a Transwell® co-culture system with the different tumor cell lines placed in the wells and naïve BM myeloid cells in the insert, with a small pore-size membrane such that only soluble factors could be transferred between the two compartments. After 48 h of incubation we found that the metastatic 4T1 cell line increased the expression of CD79a on BM myeloid cells whereas the non-metastatic 4T07 or 67NR cell lines had little effect (Figure 4A). Secreted factors from the metastatic 4T1 cells also induced the selective expansion of CD79a expressing myeloid cells (Figure 4B), and enhanced their migration (Figure 4C). The CD79a+ myeloid cells were intrinsically more migratory than the CD79- cells in response to factors secreted by the 4T1 cells. Based on these results we concluded that soluble factors secreted by the metastatic cells induce the expression of CD79a on immature myeloid cells of BM origin and modify their phenotype. To try to identify these factors, conditioned medium from the metastatic 4T1 and the non-metastatic 67NR cell lines was analyzed for differential expression of candidate cytokines using Aushon Protein Arrays. Several cytokines were found to be significantly more highly expressed by 4T1 compared to 67NR cells (Supplemental Table S1). These included GM-CSF, IL-6, and IL-1β. However, none of these individual cytokines showed any effect on CD79a expression in the naïve BM cells (data not shown), and TGF-β, G-CSF and M-CSF were also tested and shown to be ineffective. Thus either some other as yet unidentified factor is involved, or the upregulation of CD79a requires the combined activity of multiple factors.

### Stimulation of naïve BM myeloid cells through CD79a enhanced their migration, their granulocytic phenotype and their suppressive effect on T cell proliferation

**Figure 5:**
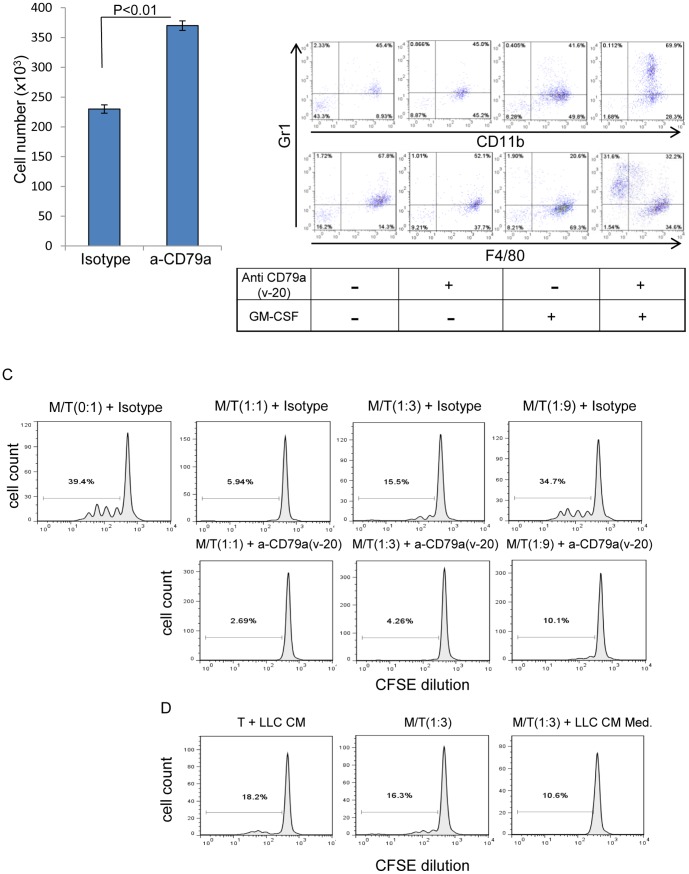
CD79a activation on myeloid cells affects migration, differentiation and T-cell suppression. (A) The effect of stimulation with anti CD79a(v-20) on the migration of FACS-sorted Ly6C+ immature myeloid cells from naive bone marrow was tested in a Transwell® system for 48 h. Results are mean +/− SEM; n = 3 determinations. (B) Bone marrow cells from naïve SCID mice were stimulated with anti- CD79a(v-20) (5 µg/ml) for 96 h with or without added GM-CSF in low (2%) normal mouse serum. The effect of anti CD79a(v-20) on myeloid cell differentiation status was assessed using immature myeloid (CD11b^+^Gr1^+^), granulocytic (Gr1^+^) and macrophage (F4/80^+^) markers. Data shown are representative of two independent experiments. (C) The effect of immature myeloid cells stimulated with anti CD79a on proliferation of CD4 T cells. Immunopurified naïve CD4+ T cells (“T”) labeled with CSFE were stimulated with anti CD3/CD28 with the addition of the indicated ratios of FACS-sorted immature BM myeloid cells (“M”) with or without the addition of anti CD79a(v-20) antibody. Cell divisions were measured by flow cytometry analysis for CFSE dilution. Dose response for effect on T-cell division of myeloid cells added at the indicated ratio, with the addition of anti CD79a(v-20) or isotype-matched control antibody is shown. (D) The effect of LLC tumor cell conditioned medium (CM) (ratio 1/5 v/v), myeloid cells and the combination of both on T cells proliferation. Data shown are representative of two independent experiments.

To determine whether CD79a has a functional role in MDSC migration we used the polyclonal CD79a(v-20) antibody to crosslink and thus activate CD79a. *In vitro* crosslinking of CD79a enhanced significantly the migration of BM myeloid cells (Figure 5A). Further analysis of the functional role of CD79a in BM myeloid cells showed that crosslinking with anti CD79a maintained the immature granulocytic phenotype (CD11b^+^Gr1^+^) while preventing differentiation toward a macrophage phenotype when the myeloid cells were co-cultured with GM-CSF (Figure 5B). One of the main characteristics of MDSCs is their ability to suppress anti-tumor T cell activity [1], so we next tested whether crosslinking CD79a has an effect on inhibition of T cell proliferation by BM myeloid cells. To that end, sorted splenic T cells were labeled with CFSE and were stimulated with anti-CD3/CD28 in the presence of different ratios of naïve myeloid cells from SCID mice with the addition of anti CD79a(v-20) or isotype control. As others have demonstrated, we found that BM-derived myeloid cells have a natural ability to suppress T cell proliferation, and this effect is dose-dependent (Figure 5C). However, the suppressive effect of myeloid cells on T cell proliferation was further increased when the myeloid cells were stimulated with anti CD79a(v-20) (Figure 5D). In addition we showed that conditioned medium from LLC tumor cells alone had a suppressive effect on T cell proliferation, but the combination of myeloid cells and tumor conditioned medium resulted in a greater suppressive effect (Figure 5C), again suggesting the possibility that a tumor-secreted factor can activate MDSCs through CD79a.

### Stimulation of myeloid cells through CD79a induces the secretion of tumor-promoting cytokines and activation of downstream signaling pathways

**Figure 6:**
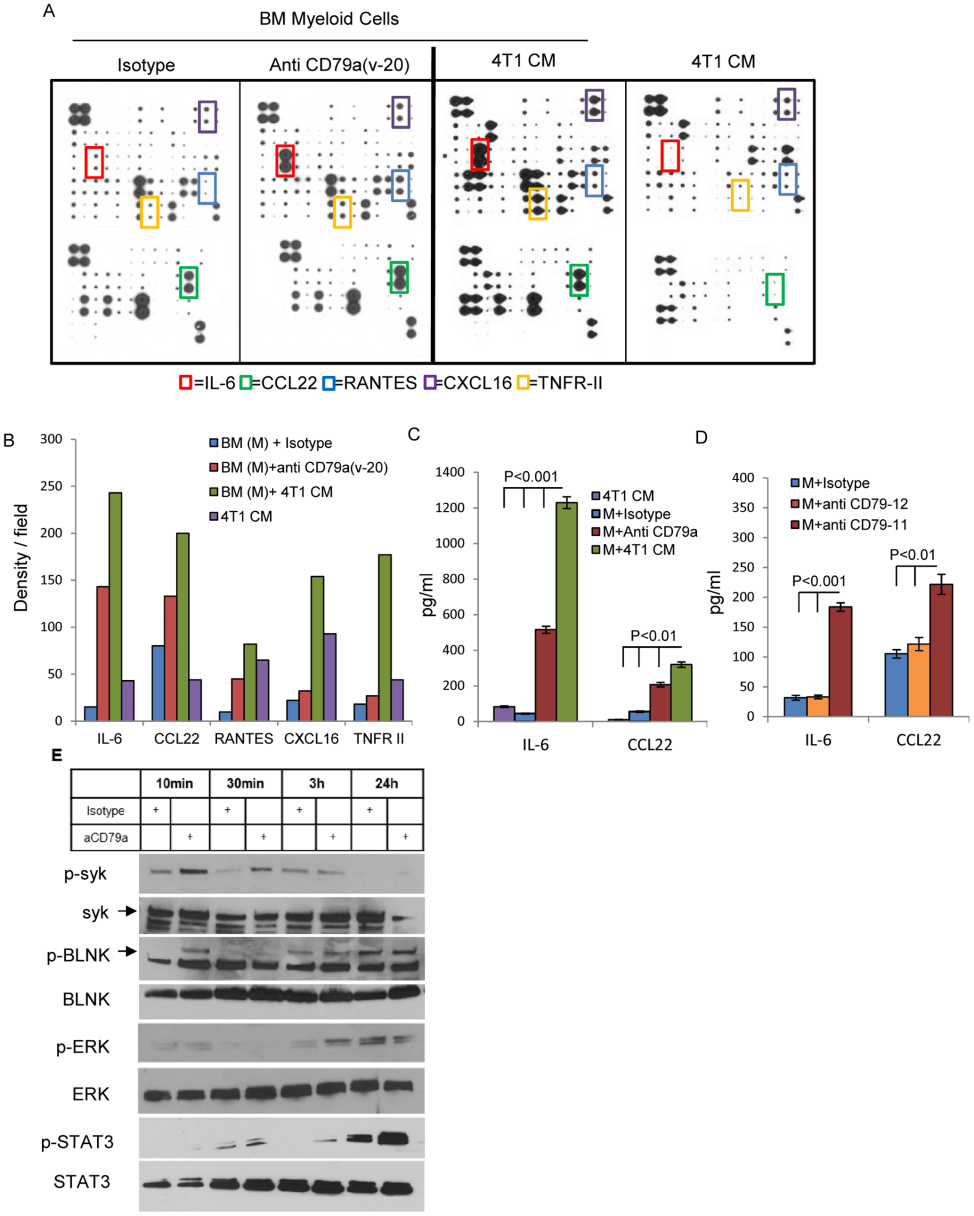
CD79a activation on myeloid cells alters cytokine expression and activates signaling pathways. The secretion of cytokines and chemokines by Ly6C^+^ immature BM myeloid cells isolated from SCID mice and cultured for 48 h under the indicated conditions was assessed using a membrane-based cytokine array. (A) Arrays of conditioned media from culture of BM cells treated with anti- CD79a(v-20), isotype control, or 4T1 tumor cell CM, together with a control array for 4T1 CM alone. (B) Relative levels of proteins with the most significant change (indicated by boxes in A) were quantitated by densitometry. (C, D) Validation of changes in IL-6 and CCL22 by Quantikine ELISA. Both the arrays and the ELISA are representative of 2 independent experiments. (E) Induction of downstream signaling events triggered by cross-linking CD79a with anti CD79a(v-20) on BM cells from SCID mice was assessed by western blot.

To further address the effect of stimulation of CD79a on the phenotype of the immature myeloid cells, we performed cytokine protein arrays using supernatants from co-culture of BM myeloid cells with anti CD79a(v-20), isotype control or 4T1 condition media (CM). We also performed a control array for the 4T1 CM alone. Stimulation of BM myeloid cells by crosslinking CD79a induced the secretion of several cytokines associated with tumor and metastasis promotion, in particular IL-6, RANTES, TNFR-II, CXCL16 and CCL22 (Figure 6A,B). A similar pattern was seen on stimulation of the BM myeloid cells with 4T1-conditioned medium, suggesting as above that conditioned medium from metastatic cells contains a factor that can activate CD79a. The increased secretion of IL-6 and CCL22 was confirmed by ELISA (Figure 6C,D). Both IL-6 and CCL22 were previously implicated in promoting tumorigenesis, by inducing expansion of immature myeloid cells and recruitment of T_reg_ respectively [34], [35].

B-cell receptor signaling through the CD79a/b heterodimer involves phosphorylation of the ITAM domains of CD79a/b leading to recruitment and activation of the kinase Syk, formation of a signaling complex around the protein BLNK, and activation of downstream pathways [21]. To explore possible signaling mechanisms induced by activation of CD79a, BM myeloid cells were stimulated with anti CD79a(v-20) and protein was extracted at different time points. Crosslinking CD79a in BM myeloid cells induced an early phosphorylation of Syk and BLNK (Figure 6E), suggesting that some of the same downstream pathways may be activated by CD79a in myeloid cells and in B-cells. Later phosphorylation of ERK and STAT3 was also observed (Figure 6E), with STAT3 activation possibly reflecting autocrine stimulation by the increased IL-6 secretion that occurs on CD79a activation (Figure 6D).

### CD79a expressing myeloid cells promote tumor growth both at the primary and the metastatic site

**Figure 7:**
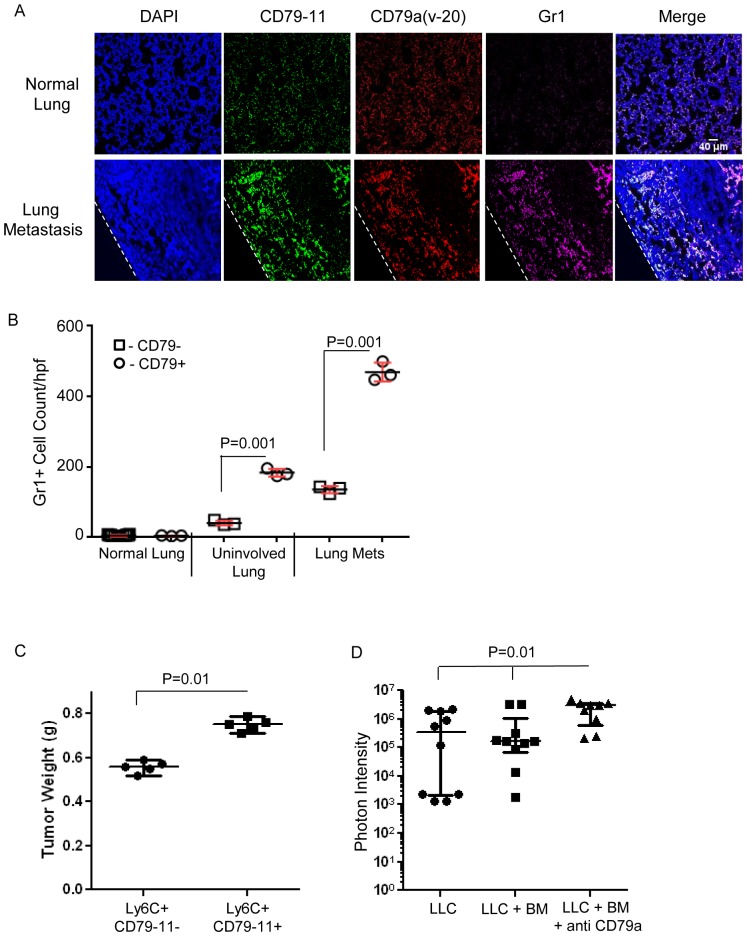
CD79a expression and activation on myeloid cells contributes to their pro-tumorigenic effects *in vivo*. (A) Immunofluorescence of normal lung and metastasis-bearing lungs in the LLC model. Infiltrating Gr1+CD79a+ myeloid cells are clearly visible at the outer edge of a lung metastasis but are rare or absent from normal lung tissue. The dotted line marks the outer boundary of the metastasis. Scale bar: 40 µm. (B) Quantification of lung infiltrating Gr1+CD79a+ and Gr1+Cd79-11- myeloid cells: Analysis was done for 3 groups: normal lung, uninvolved lung tissue from metastasis bearing lung, and lung metastasis. Each data point represents the mean of 5 fields (500 µm^2^) for a given sample and results are given as mean +/− SEM. (C) Immature BM cells from C57Bl/6 mice were FACS sorted into two groups of Ly6C^+^CD79a^+^ and Ly6C^+^ CD79a^−^ myeloid cells. The sorted myeloid cells were co-inoculated together with LLC tumor cells subcutaneously in C57Bl/6 mice at a ratio of 25∶1 myeloid: tumor cells. Tumor weight was measured at day 14 post-inoculation. Results are mean +/− SEM (n = 5). (D) Ly6C+ BM-derived myeloid cells from naïve mice were isolated by FACS and cultured with anti CD79a(v-20) or isotype control antibodies for 24 h. Treated myeloid cells were then harvested and injected i.v. together with luciferase-expressing LLC cells (2×10^6^ myeloid cells and 10^4^ LLC tumor cells/mouse) in C57Bl/6 mice. Metastatic burden was quantitated from *in vivo* luciferase signal at day 15 after implantation. Results are median with interquartile range (n = 10 mice/group).

MDSCs have previously been shown to infiltrate primary tumors and metastases [1]. By immunofluorescence, we showed that the infiltrating MDSCs in metastases from the LLC model co-express the myeloid marker Gr1 together with CD79a, as detected with either anti CD79-11 or anti CD79a(v-20) antibodies (Figure 7A). Quantifying the images, we confirmed that the lung metastases had significantly higher levels of total MDSC infiltration when compared with non-involved areas of the metastasis-bearing lungs, or with naïve lungs, and we showed that the majority of the MDSCs in the metastases and the uninvolved lung from tumor-bearing mice were CD79a+ (Figure 7B). The contribution of CD79a to the tumor-promoting effect of myeloid cells was assessed in two ways. To elucidate the role of CD79a-expressing myeloid cells on primary tumor formation, two myeloid cell populations (Ly6C^+^CD79a^−^ and Ly6C^+^ CD79a^+^) were sorted from BM cells of 4T1-tumor bearing SCID (Balb/c) mice harvested 20 days after tumor cell innoculation. Cells from either population were co-inoculated together with 4T1 tumor cells into the mammary fat pad of immune competent mice, and tumor weight was evaluated at 14 days post inoculation. The CD79a^+^ myeloid cells caused a significantly greater stimulation of tumor growth (Figure 7C). To examine the role of CD79a ^+^ myeloid cells in lung metastasis, Ly6C^+^ myeloid cells were sorted from bone marrow of naïve C57Bl/6 mice and incubated *ex vivo* with anti CD79a(v-20) or isotype control antibody for 24 h. Thereafter myeloid cells were co-injected together with luciferase-expressing LLC cells into the tail vein of syngeneic mice. Lung metastasis burden was assessed by luciferase imaging after 21 days, and the myeloid cells stimulated with anti-CD79a(v-20) significantly enhanced metastasis compared with unstimulated myeloid cells (Figure 7D). Together, these data suggest that activation of CD79a on myeloid cells contributes to the promotion of tumorigenesis at the primary and metastatic sites.

### CD79a is expressed on myeloid BM cells from normal human donors and is upregulated on myeloid cells circulating in blood from cancer patients

**Figure 8:**
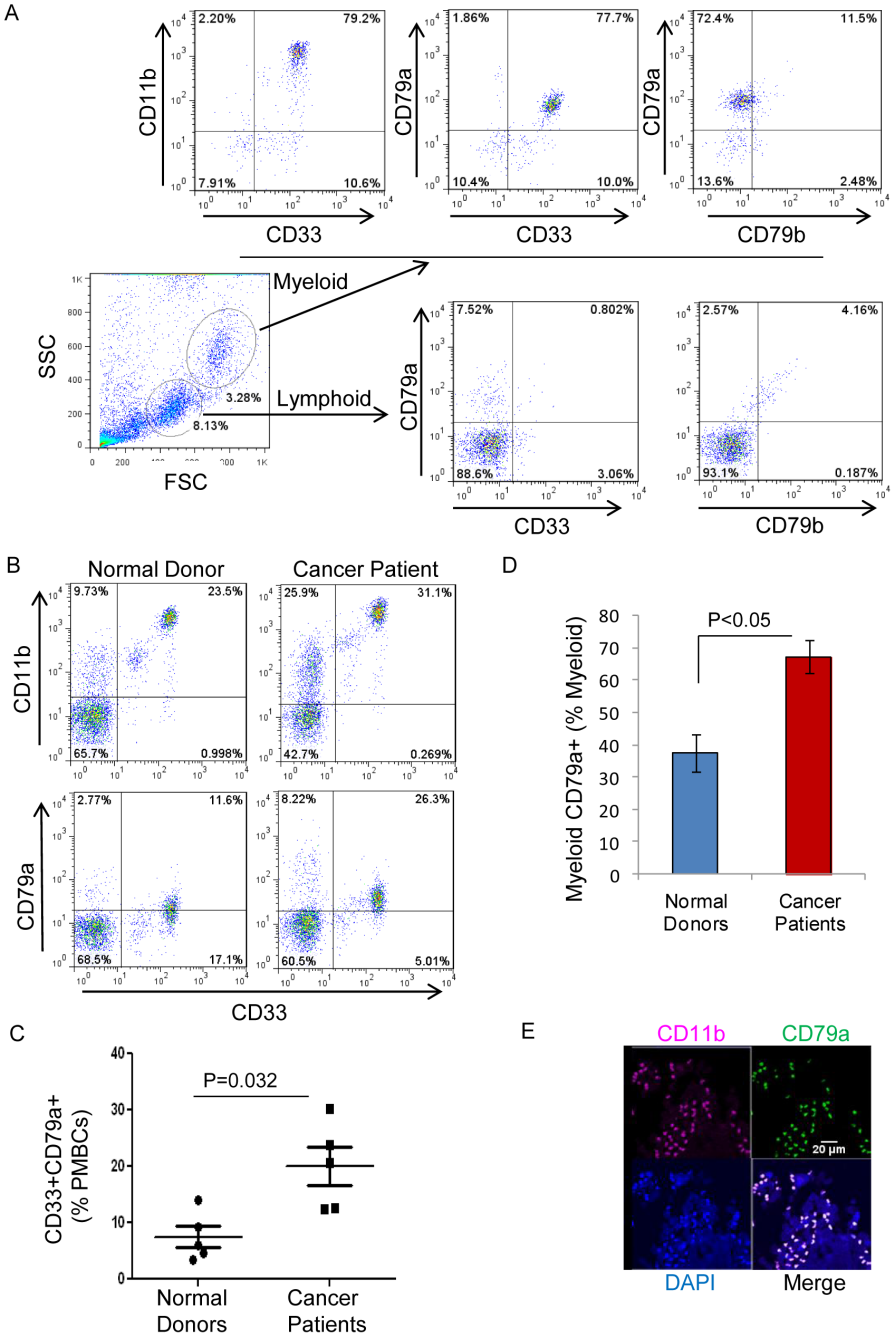
CD79a expression on myeloid cells from normal human donors and in cancer patients. (A) Expression of CD79a on immature myeloid cells (characterized as CD11b^+^CD33^+^) in BM from a normal human donor. (B) Representative FACS profiles of immature myeloid cells in peripheral blood (live leukocyte gate) from a normal donor and a lung cancer patient. (C) Immature myeloid cells (CD11b^+^CD33^+^) expressing CD79a in normal donors and lung cancer patients as a% of live leukocytes. Results are mean +/− SEM (n = 5). (D) Histogram showing proportion of CD79a+ myeloid cells relative to total myeloid cells. Results are mean +/− SEM (n = 5) (E) Representative immunofluorescence staining of infiltrating CD79a^+^ myeloid cells in a human invasive breast carcinoma. Scale bar; 20 µm.

The data thus far support the hypothesis that in mouse models of metastatic disease, the tumor can increase expression of CD79a on immature myeloid cells, thereby maintaining a more immature phenotype with immunosuppressive and tumor promoting characteristics. We next wanted to know whether CD79a is expressed on myeloid cells in humans. We found that CD79a is expressed on immature BM-derived myeloid cells from normal human donors (Figure 8A), as was seen in mice. Importantly we found that CD79a was significantly upregulated on myeloid cells in peripheral blood from lung cancer patients in comparison to normal donors (Figure 8B,C,D). Furthermore, immunofluorescence staining of human breast tumor sections showed tumor infiltration by myeloid cells (CD11b+) that express CD79a (Figure 8E). 34% of the breast cancer samples examined (n = 60 total) were positive for CD79a^+^ infiltrating myeloid cells. Information was not available on whether these patients had distant or only local disease. Thus CD79a expression on immature myeloid cells and MDSCs is seen in both humans and mice. However, it will be important to determine in humans whether elevated CD79a expression on myeloid cells correlates with the metastatic state, as it appears to in mouse, and whether it might be useful as a prognostic marker.

## Discussion

**Figure 9:**
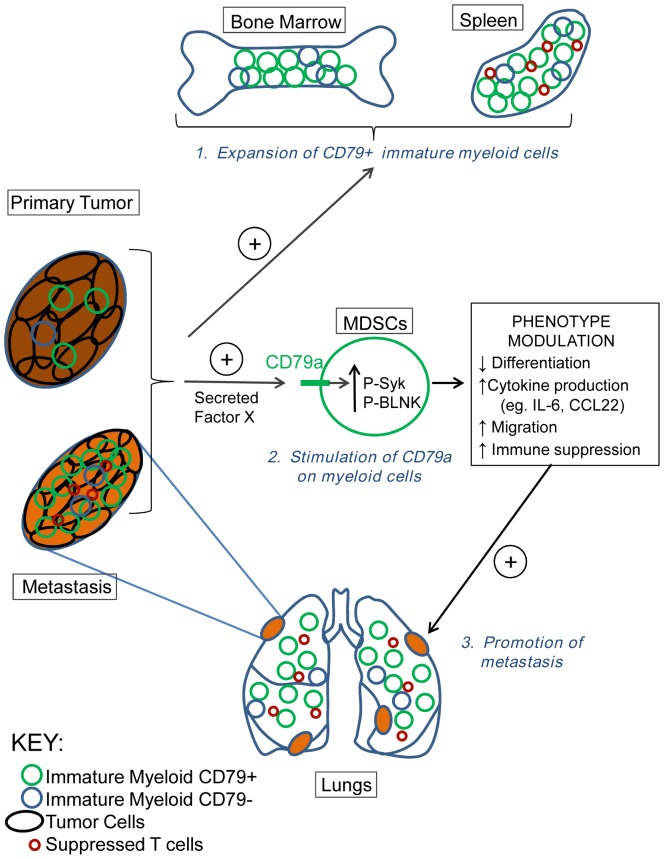
Role for CD79a in reciprocal interactions between immature myeloid and tumor cells that promote tumorigenesis and metastasis. Metastatic tumors induce the expansion of an immature myeloid cell population in bone marrow and spleen, and drive enrichment of a subpopulation that expresses elevated CD79a. An as yet unidentified CD79a ligand secreted by tumor cells activates the CD79a on the immature myeloid cells leading to signaling through Syk and BLNK and modulation of cellular phenotype in a variety of ways. These include maintainance of the immature state, upregulated expression of tumor-promoting cytokines and chemokines, increased migration, and enhanced immunosuppressive activity. This phenotypic modulation induces and/or amplifies the stimulatory effect of MDSCs on tumorigenesis and metastasis.

The best-characterized role of CD79a is as part of the B cell receptor signaling complex. CD79a expression is seen very early in B cell lineage development in bone marrow, and it has an essential role in B cell development, survival and activation [23], [36]. In the present study we unexpectedly found expression of CD79a on immature BM-derived myeloid cells, and on tumor-induced MDSCs in multiple mouse tumor models and in human cancer patients. Most importantly, activation of CD79a on MDSCs enhanced tumorigenesis and metastasis in the mouse models, suggesting a functional role for myeloid CD79a in promoting tumor progression. Mechanistically, we showed that stimulation of MDSCs through CD79a maintained their immature status, enhanced their suppressive effect on T cell proliferation, stimulated their migration, and induced the secretion of pro-tumorigenic cytokines (see schematic in Figure 9).

CD79a expression on myeloid cells was first reported in some cases of acute myeloid leukemia (AML) which showed co-expression of CD79a with myeloid markers [26], [27]. In these studies the proportion of myeloid cells that co-expressed CD79a ranged from 0–90% depending on the study. Exploring this heterogeneity, Bhargava and colleagues showed by immunohistochemistry that the detected level of CD79a on myeloid cells is dependent on the antibody clone used, revealing 30–45% AML cases positive for CD79a using the clones 11D10 and HM57 [37]. The highest frequency of CD79a expression was detected using antibodies specific for the intracellular domain. Interestingly, in the same study they also found CD79a expression on 30–40% of normal myeloid precursors in the early stages of maturation, while bands and mature neutrophils did not stain with these antibodies. However, the authors raised the possibility that the apparent expression of CD79a on normal immature myeloid cells might be an immunohistochemical artifact.

In the present study we showed by FACS-based immunophenotyping that CD79a was expressed on the majority of naïve BM myeloid cells, as well as on a small population of peripheral myeloid cells in all the mouse models that we examined. Since there is no good monoclonal Ab for the extracellular domain of CD79a, we used a monoclonal Ab (clone HM79-11a) described as reactive with the dimer CD79a/b. By this approach, we found that immature BM myeloid cells were positive for CD79a/b (detected with HM79-11), but not for CD79b (as detected with the CD79b-specific clone HM79-12), or for other B cell markers. Similar results were found using a polyclonal Ab generated against the extracellular domain of CD79a (CD79a-poly), though staining with this antibody was much weaker. CD79a expression on immature BM myeloid cells from SCID mice was further confirmed by intracellular staining using clone F11-172. The expression of CD79a but not CD79b in immature myeloid cells was also confirmed at the mRNA level in BM myeloid cells from SCID mice, which lack the lymphoid compartment. By Western blots analysis, CD79a in MDSCs had a molecular weight of 37 KDa, slightly lower than the lowest band seen in B cells. CD79a is known to exist as multiple forms due to alternative splicing and glycosylation variants [38], and it will be interesting to characterize this myeloid isoform further. Overall, our flow cytometry results using multiple antibodies and our gene expression data are in accordance with the immunohistochemical analysis of Bhargava et al. [37], and confirm that CD79a is expressed early in the myeloid lineage. Similar to our results in the mouse models, we found that normal BM and a fraction (10–15%) of circulating myeloid cells from normal human donors expressed CD79a. Together, these observations suggest that CD79a may play a role in early myeloid development, and not be restricted to the B-cell lineage in normal hematopoiesis. It is intriguing that one alternate model of hematopoietic cell diversification and development proposes that commitment to the B cell and T cell lineages occurs through myeloid/B cell and myeloid/T cell bipotential stages [39], [40]. This contrasts with the classical model in which T and B cells develop from a common lymphoid progenitor after initial separation of distinct myelo-erythroid and lymphoid lineages. Conceivably the expression of CD79a that we observe on immature myeloid cells may reflect an early stage in the diversification of myeloid cells and B cells from such a bipotential myeloid/B cell progenitor. However, the functional role of CD79a in normal immature myeloid cells is not clear, as to date no abnormalities in myelopoiesis have been described in the CD79a knockout mouse [41].

In normal conditions and in acute inflammation, immature myeloid cells rapidly undergo differentiation into the different mature myeloid cells (macrophages, granulocytes or dendritic cells) either in the BM, or following recruitment to the periphery. However in chronic inflammation and cancer there is aberrant expansion of certain immature myeloid populations, including MDSCs [1]. Since we found that MDSCs expanded by metastatic tumors maintain the CD79a expression seen in immature BM cells, we asked whether CD79a plays a role in myeloid differentiation. Knockout mouse studies have shown that both CD79a and CD79b are necessary for differentiation of pro-B to pre-B cells in response to antigen engagement of the BCR [41]. However, dimers of the CD79a/b or of CD79a/a cytoplasmic domains alone can induce tonic antigen-independent signaling in B cell progenitors to support early stage differentiation 42,43. Furthermore, cross-linking of CD79a in early lineage B-cells was sufficient to induce downstream tyrosine phosphorylation though the functional consequences were not explored [44]. Here we showed that BM myeloid cells activated by CD79a cross-linking maintained their immature phenotype (CD11^+^Gr1^+^F4/80^−^) on treatment with GM-CSF, whereas GM-CSF alone promoted the differentiation toward the F4/80+ macrophage phenotype. Since we did not find CD79b on immature myeloid cells, our data suggest that CD79a can function independently of CD79b to generate signals that maintain the immature state in cells of the myeloid lineage.

We also observed other important consequences of CD79a activation that could contribute to the pro-tumorigenic effect of the MDSCs. Cross-linking CD79a enhanced MDSCs migration, a finding that is in accordance with our immunofluorescence observations showing that that a large proportion of MDSCs infiltrating into lung metastases express CD79a. Furthermore, cross-linking CD79a on MDSCs also increased their inhibitory effect on T cell proliferation and changed their cytokine expression profile. The cytokines that were most significantly upregulated following CD79a stimulation of MDSCs were IL-6 and CCL22. IL-6 is a major activator of STAT3 signaling and it is associated with poor prognosis in different cancers [45], [46]. IL-6 was found to be involved in tumor growth and metastasis spread by inducing expansion of MDSCs and immune suppression [34], [47], and it can also affect the tumor parenchyma by inducing a more malignant, cancer stem cell phenotype in breast cancer cells [48], [49]. CCL22 is a major recruiter of T_regs_, which support the immune suppression role of MDSCs [35]. Thus secretion of these two cytokines by MDSCs following stimulation through CD79a could clearly enhance the pro-tumorigenic activity of these cells.

Currently the endogenous ligand for CD79a is not known, and it is not clear how the activated CD79a signals to induce pro-tumorigenic responses in MDSCs. As an ITAM-bearing protein, CD79a joins an increasing family of myeloid adaptor molecules and receptors that use ITAMs as part of their signaling mechanism [50], [51]. ITAM-containing signaling adaptors are generally associated with activation of cellular responses, though some ITAM adaptors in myeloid cells can have an inhibitory role [52]. Here we showed that co-culture of MDSCs with metastatic tumor cells, or exposure of MDSCs to tumor cell-conditioned medium, sustained the expression of CD79a in MDSCs in culture, and induced many of the same responses as did activation of CD79a by the anti-CD79a antibody. Thus our data strongly suggest that a factor secreted by tumor cells may be responsible for these activities. However, when we tested the candidate cytokines that were most differentially expressed between the metastatic 4T1 and non-metastatic 67NR cells (GM-CSF, G-CSF, M-CSF, IL-6, IL-1β, TNFα), we did not find any with the expected activity on MDSCs, so the identity of the secreted factor remains unknown. CD79a has a very short extracellular domain, making it unlikely that it engages ligand directly. Increasing evidence suggests that ITAM-bearing adaptor molecules can interact with many different classes of receptor for signaling, including toll-like receptors, tumor necrosis factor receptors, cytokine receptors that use the Jak-STAT signaling pathway, and integrins [53]. Interestingly, myeloid cells have a large number of C-type lectin receptors that recognize damaged and aberrant cells, and some of these receptors are dependent on ITAM adaptor proteins for signaling [54]. Finding the ligand/receptor pair that interacts with CD79a will be important for elucidating the role of CD79a in myeloid cells.

In B-cells, engagement of the B-cell receptor leads to phosphorylation of the CD79a/b heterodimer and consequent recruitment and activation of the tyrosine kinase Syk [21]. Syk activation organizes two signaling complexes which activate secondary messenger pathways including the Ras/ERK, NFAT and NF-kB pathways, ultimately leading to altered cytoskeletal organization and changes in gene expression [21]. Here we found that cross-linking CD79a in immature BM myeloid cells resulted in early Syk phosphorylation. Thus downstream signaling from CD79a in myeloid cells may involve some of the same players as seen in B-cells. CD79a is unique among ITAM-bearing proteins, and differs importantly from CD79b, in having an additional tyrosine (Y204) outside the ITAM motif that is critical for B-cell activation and proliferation. In B-cells, phosphorylation on this site recruits BLNK which nucleates the signaling complex that activates the Ras/ERK pathway [55]. We did observe an increase in BLNK phosphorylation on stimulation of CD79a, and it will be interesting to determine if this unique phosphorylation site on CD79a is key to the recruitment of downstream mediators in the myeloid cells. We also observed a later STAT3 phosphorylation that probably reflected the establishment of an IL-6 autocrine loop following CD79a stimulation. STAT3 activation has previously been implicated in promoting increased survival and proliferation of myeloid progenitor cells, as well as in blocking their differentiation [1].

In summary, we have demonstrated expression of the B cell receptor subunit, CD79a, on immature myeloid cells and MDSCs in multiple mouse models of cancer and different mouse strains. CD79a was found also on normal human immature BM myeloid cells and upregulated on peripheral MDSCs from cancer patients. We have provided evidence that CD79a activation by tumor-derived factors contributes importantly to maintaining the immature phenotype in myeloid cells and to enhancing their immune suppressive and pro-tumorigenic activities. A number of strategies to target MDSCs are currently being explored in the field, including induction of differentiation with agents such as all-trans retinoic acid; inhibition of expansion by targeting factors such as SCF and VEGF; and inhibiting function with agents such as COX2 inhibitors [1]. With our discovery of a functional role for CD79a in the tumor suppressive effects of MDSCs, it will be interesting to determine whether targeting CD79a or downstream signaling events would add to this arsenal of anti-MDSC approaches. Drugs such as fostamatinib, an inhibitor of the Syk kinase that has shown some clinical activity in non-Hodgkin lymphoma and chronic lymphocytic leukemia [56],[57], could conceivably be repurposed to provide therapeutic benefit in solid tumors. While a recent Phase I trial did not show any benefit of fostamininib monotherapy in heavily pre-treated patients with solid tumors [58], understanding the role of CD79a signaling and Syk kinase in MDSCs may prompt investigation of Syk kinase inhibitors as immunomodulatory agents in combination therapy protocols. Further study is warranted to elucidate this new signaling pathway in MDSCs and its modulation by tumor-derived factors.

## Materials and Methods

### Cell lines

The malignant cell lines 67NR, 4T07, and 4T1 were all established from a single spontaneously arising mouse mammary tumor in a BALB/c mouse [28], and were the generous gift of Dr. Fred Miller, Karmanos Institute, Detroit, MI. The Lewis lung carcinoma (LLC) cells stably expressing a luciferase-GFP fusion protein were obtained from Dr. Chi-Ping Day, NCI [59]. Cells were cultured in DMEM/10% fetal bovine serum.

### Mice and tumorigenesis studies

All animals studies were performed under a protocol (LC-070) approved by the National Cancer Institute Animal Care and Use Committee, in accordance with Association for Assessment and Accreditation of Laboratory Animal guidelines and policies established by the NIH. Balb/c, C57Bl/6 and SCID (Balb/c) mice for tumor implantation and immunophenotyping of naïve mice were purchased from Charles River, Frederick, MD and were used at 8–10 weeks old. Two genetically engineered mouse models of breast cancer were also used. The MMTV-PyMT model (FVB/N background) is highly metastatic to the lung [60], and was obtained from Dr. Kent Hunter, NCI. The BrCaco/co;MMTV-Cre; Trp53+/− model (“BrCa1” on mixed C57Bl/6, FVB/N background) forms large primary tumors but is rarely metastastic [61], and was obtained from Dr. Chuxia Deng (NIDDK). Tumor-bearing MMTV-PyMT mice were analyzed at 3 months of age at which time >90% of the mice had developed lung metastases, while tumor-bearing BrCa1 mice were analyzed at 6 months of age. For syngeneic transplant studies, the cell lines 4T1, 4T07, (4×10^4^ cells/mouse) and 67NR (6×10^4^ cells/mouse) were surgically implanted in the number 4 mammary fat pad of Balb/c mice. The Lewis Lung (LLC) cell line was implanted (4×10^4^ cells/mouse) subcutaneously on the flank of wildtype C57Bl/6 mice. Tumor growth was measured by calipers and tumor volumes were calculated as v = 0.52*L*S*S, where L is the longest diameter and S is the orthogonal shorter diameter. Tumor-bearing mice were euthanized 3–5 weeks post-inoculation. At necropsy, lungs were inspected for the presence of gross metastases and the largest lobe was fixed in neutral buffered formalin for histopathological assessment of metastatic burden and for localization of infiltrating immune cells by immunofluorescence. The remaining lung lobes were used for immunophenotyping. Bone marrow and peripheral blood were also collected for immunophenotyping (see later), and primary tumors were fixed in formalin. To assess the role of CD79a^+^ myeloid cells on primary tumorigenesis, Ly6C^+^CD79-11^+^ and Ly6C^+^CD79-11^−^ myeloid cells were prepared by FACS sorting from bone marrow of 4T1-tumor-bearing SCID mice harvested 20 days after tumor cell implantation. The myeloid cells were orthotopically co-implanted with 4T1 tumor cells in immunocompetent Balb/c hosts (1×10^6^ myeloid cells: 1×10^4^ tumor cells), and the weight of the primary tumor was determined after 14 days. To assess the role of CD79a^+^ myeloid cells in metastasis, 1×10^4^ luciferase-labelled LLC cells were injected into the tail vein of immunocompetent C57Bl/6 mice, either alone, or with 1×10^6^ myeloid cells that had been FACS-sorted from bone marrows of naïve C57Bl/6 mice and treated *ex vivo* for 24 h with either anti-CD79a(v-20) or isotype control antibody (10 µg/ml). The lung metastatic burden at the endpoint was determined by *in vivo* bioluminescent imaging following intraperitoneal injection of 150 mg/Kg luciferin substrate using a Xenogen IVIS system (Caliper Life Sciences, Alameda, CA), and confirmed by histopathological analysis of H&E stained sections of formalin-fixed lung.

#### Human clinical specimens

Studies with human clinical material were done under an NIH Office of Human Subjects Research exemption (OHSRP 11416) that allows for the use of de-identified specimens for biological research. Fresh blood from normal donors was obtained from the Blood Bank at the National Institutes of Health Clinical Center, Bethesda. Anonymous blood samples from consented human lung cancer patients were obtained from the *Cooperative Human Tissue Network* (*CHTN*) and shipped overnight in EDTA tubes on ice for analysis the next day. Patients tumor grade was 1–3, and all patients were receiving standard-of-care treatment. Offcuts from a previously-described breast cancer tissue microarray (TMA) of formalin-fixed paraffin-embedded invasive breast cancers from Polish patients [62], were used for immunofluorescence staining of myeloid and B-cell markers as described later. Since the TMA sections were cut from towards the end of the block, many cores were missing or mislocalized and so remaining cores could not be reliably associated with patient information. Thus immunostaining scores could not be associated with clinicopathologic parameters, but did allow for an assessment of overall frequency of a given staining pattern within the population.

### Preparation of single cell suspensions for flow cytometry and sorting

Single cell suspensions for flow cytometry and FACS were prepared in complete RPMI 1640 medium containing 10% heat-inactivated Fetal Bovine Serum (FBS) (GIBCO), 50 µM 2-mercaptoethanol and penicillin/streptomycin (GIBCO). Lungs were perfused with PBS to remove cells in the circulation prior to processing. Both lungs and spleens were then mashed with the plunger of a 1 ml syringe through a 70 µm cell strainer (BD Falcon). BM cells were isolated by flushing femurs with complete RPMI 1640 medium. Single cell suspensions from the different organs were washed with ice cold PBS, and any remaining red blood cells were lysed in ACK Lysis Buffer. Mouse blood was obtained by cardiac puncture. Blood samples from both mouse and human were lysed twice in ACK buffer. Cells were recovered in complete medium, filtered through 40 µm cell strainers, counted with a Cellometer (Nexcelom Bioscience, MA) and maintained on ice for further analysis.

### Flow cytometry and FACS sorting

Cells surface Fc receptors were blocked by incubation with anti-Fc (CD32/CD16, clone 93) (BioLegend, CA) for 15 min. Mouse cells were stained with the relevant antibodies in FACS buffer for 30 min at 4°C, washed and stained with 7-AAD to enable exclusion of dead cells. For intracellular staining, cells were fixed in paraformaldehyde 2% for 15 min, washed and permeabilized with Saponin 0.01% and stained with the relevant antibodies. Anti-mouse antibodies used for FACS staining (BioLegend, CA) were the following: CD11b (M/170), Gr1(RB6-8C5), IgM (MM-30), CD19 (6D5), B220 (30-F11), Ly6C (HK1.4), Ly6G (1A8), F4/80 (BM8). For mouse studies, the antibodies, anti-CD79a and CD79b used for flow cytometry and/or for *in vitro* cell stimulation are described in table 1.

**Table 1:** The following anti-CD79a and CD79b antibodies were used in the mouse studies for flow cytometry and/or for *in vitro* cell stimulation.

Antibody	Clone	Antigen	Source	Catalog #
CD79-11	HM79-11	CD79a/b	AbD Serotec (Raleigh, NC)	MCA1821FB
CD79-12	HM79-12	CD79b	BioLegend (CA)	132804
CD79a	F11-172	CD79a intracellular	BioLegend (CA)	133106
CD79a(v-20)	(polyclonal)	CD79a extracellular peptide	Santa Cruz Biotechnology (CA)	sc-8503

For FACS analysis of human samples the following antibodies (eBiosciences, CA) were used: CD11b (CBRM1/5), CD33 (HIM3-4), CD79b (CB3-1), CD19 (H1B19). Anti CD79a (ZL7-4) was purchased from AbD Serotec, NC. Flow cytometry was performed on a FACSCalibur (BD Biosciences, CA) instrument and analyzed with FlowJo software (FlowJo, OR). FACS sorting was performed on a FACS Aria (BD Biosciences, CA). FACS analysis was done by gating on leukocytes using forward scatter and side scatter, followed by gating on live cells (negative for 7-AAD).

### Quantitative real-time PCR (qRT-PCR)

For analysis of CD79a and CD79b gene expression, BM cells were isolated from SCID mice and FACS sorted for Ly6C+ myeloid cells. Splenocytes from naïve Balb/c mice were used as a positive control. Total RNA was prepared using TRIzol (Invitrogen) until the step of collecting the aqueous fraction. Total RNA was then extracted from the aqueous fraction using the RNeasy kit (Qiagen Inc, Valencia CA, USA) according to the manufacturer's instructions. cDNA synthesis was carried out using M-MLV reverse transcriptase (Invitrogen). All quantitative RT-PCR data were normalized to cyclophilin A (PPIA) as an internal control for each sample. The following primers were used: CD79a Forward, (5′- CAGTCAAGGTTCAGGCCCTCAT -3′); and Reverse, (5′- CCTGTTTGGGTCCCGCATGCC -3′); CD79b, Forward (5′- GCTCTTCTCAGGTGAGCCGGT-3′), Reverse (5′- GTCTGGTACATGTTCAAGCCCTCA-3′); PPIA, Forward (5′- CATACAGGTCCTGGCATCTTGTC-3′), Reverse (5′-AGACCACATGCTTGCCATCCAG-3′).

### Western Blot

For western blot analysis of CD79a and CD79b, single cell suspensions of BM and spleen were prepared from wildtype (WT) Balb/c or C57Bl/6 mice and immunodeficient SCID mice, in the naïve or tumor-bearing state. Bone marrow cells were pooled from 5 mice. Cells were lysed in Mem-PER buffer (Pierce). Total protein (40 µg) was electrophoresed on 4 to 20% Tris-glycine gels and transferred to polyvinylidene fluoride membranes. Membranes were blocked with 5% nonfat dry milk in Tris-buffered saline with 0.1% Tween-20 for 45 minutes at room temperature, and were then incubated with primary antibody solutions overnight at 4°C. The primary antibodies used were as follows: anti-CD79-11 (HM79-11, AbD Serotec), anti-CD79b (HM79-12, BioLegend, CA), anti-CD79a (F11-172, BioLegend, CA), anti-β-actin (Sigma). For detection of signaling components, the following antibodies (Cell Signaling Technologies, Beverly, MA) were used: anti-Syk (clone D1I5Q), anti-phospho-Syk (clone C87C1), anti-ERK1/2(p42/p44, 9102), anti-phospho-ERK (clone E10), anti-STAT3 (clone 79D7) and anti-phospho-STAT3 (clone D3A7). Anti-BLNK (polyclonal, bs-2748R) and anti-phospho-BLNK (polyclonal, bs-3054R) were purchased from Bioss Inc, Woburn, MA. All secondary antibodies were HRP-conjugated goat anti-Armenian hamster (Rockland, PA), used at a 1∶1,000 dilution. Secondary antibodies were applied for 2 hours at room temperature, followed by incubation with chemiluminescent reagent (Super Signal; Thermo Scientific Pierce Protein Research Products, Rockford, IL, USA) and exposure to autoradiography film (Eastman Kodak Co., Rochester, NY, USA).

### Suppression of T cell proliferation

To test the effect of MDSCs on suppression of T cell proliferation we used the carboxy-fluorescein-diacetate-succinimidyl-ester (CSFE) labeling assay [63]. T cells were negatively isolated by separation with magnetic beads (Pan T Cell Isolation Kit; Miltenyi Biotec). T cells were suspended into RPMI-1640 medium (1×10^7^ cells/ml). CFSE (Sigma-Aldrich, St Louis, MO, USA) was added to the cell suspension at a final concentration of 5 µM and incubated for 15 min at room temperature in darkness. Labeled cells were washed 3 times with PBS, re-suspended into RPMI-1640 media and counted. Ly6C+ myeloid cells were isolated from SCID mice BM cells by FACS sorting (Aria, BD). T cells and myeloid cells were co-cultured in RPMI-1640 medium containing 1% normal mouse serum. Cells were plated in round bottom 96-well plates in triplicates at ratio of 1∶0, 1∶1, 3∶1, 9∶1 T cells/myeloid cells at a final number of 1×10^6^ cells/well. In some experiments, anti CD79a antibody (HM79-11, Serotec) was added at 10 µg/ml, or conditioned medium from 4T1 cell culture was added at 1/5 final dilution. After 5 days of culture, cells were collected, triplicate wells were pooled and cells were stained with anti-CD4 and analyzed by flow cytometry for CFSE dilution.

### Transwell-based assays to assess effects of tumor cell secreted factors on myeloid cell expression of CD79a, expansion and migration

To determine the effect of tumor cell secreted factors on CD79a expression and expansion of myeloid cells, 4T1 or 67NR tumor cells were plated (3×10^3^ cells/well) in the bottom chamber of a 24-well Transwell® system with 0.4 µm pore size insert (BD Falcon, CA). 24 h later, 3×10^6^ BM or spleen cells were placed in the insert and co-incubated with the tumor cells for 48 h in complete media containing 10% FBS. Cells in the insert were then harvested and analyzed by FACS. To assess the ability of tumor cells to influence migration of myeloid cells, the 24-well Transwell® system with 3 µm pore inserts was used (BD Falcon, CA) and cells were seeded as above. At 48 h non-attached cells were collected from the well and from the insert separately, counted and analyzed by FACS for co-expression of the myeloid markers Ly6C, Gr1 and CD11b together with CD79a. To test the effect of anti CD79a antibody stimulation on myeloid cell migration, BM or spleen cells were placed in the insert (3×10^6^ cells/insert) with or without addition of anti CD79a (10 µg/ml) to the well. After 48 h cells were collected from the well and from the insert separately, counted and analyzed by flow cytometry for co-expression of the myeloid marker Ly6C, together with CD79a.

### Cytokine protein array and ELISA

To assess cytokine secretion from mouse tumor cells, conditioned media from 67NR and 4T1 tumor cell cultures were analyzed for 14 different cytokines (see Supplementary Table S1 for list) using quantitative Aushon Protein Arrays, performed by the manufacturer (Aushon Biosystems, Billerica, MA). To assess cytokine secretion from myeloid cells, semi-quantitative dot-blot based Mouse Cytokine Arrays C1000 (RayBiotech Inc, Norcross GA) were used according to the manufacturer's instructions. After blocking with BSA, the arrays were incubated with a 1∶1 dilution in blocking buffer with supernatant from Ly6C+ BM myeloid cells of SCID mice (2×10^6^/well in 24 well plate) cultured with anti- CD79a (10 µg/ml) or isotype control for 48 h. A parallel set of arrays was used to probe cytokine expression in the supernatant from myeloid cells that had been exposed to 4T1 condition medium (at a final concentration of 20%) for 48 h. 4T1 conditioned medium alone (20% final) was used as a control. Signal was developed according to the manufacturer's protocol, and imaged by exposure of the membrane to film. Relative spot intensity was determined using Image J. The concentration of IL-6 and CCL22 in culture supernatants was quantitated by Quantikine ELISA (R&D Systems, Minneapolis, MN) according to the manufacturer's instructions.

### Immunofluorescence staining

Lungs from naïve or tumor-bearing mice were removed, inflated with 10% formalin, maintained in 10% formalin overnight, paraffin embedded and sections (5–6 µm) were prepared. Tissue sections were heated (60°C, 20 min), deparaffinized (3 washes in xylenes), rehydrated by successive washes in absolute ethanol, 90% ethanol, 75% ethanol and deionized water. Antigen retrieval was performed at 125°C for 20 min in pH 9.0-EDTA buffer. Tissue sections were incubated for 1 hour in a blocking solution (PBS, 5% goat serum, 0.05% Tween 20). Immunofluorescence staining was performed using the following primary antibodies for mouse samples: Gr1(RB6-8C5)and F4/80 (BM8) from BioLegend, CD79a (HM79-11, AbD-Serotec) and CD79a polyclonal IgG (Santa Cruz, CA). For human samples the following antibodies were used: CD11b (CBRM1/5), CD79a (ZL7-4), CD79b (CB3-1) (BioLegend, CA). Primary antibodies were incubated overnight at 4°C. Tissue sections were washed twice and stained with the following secondary antibodies: Alexa 546-conjugated donkey anti-goat, Invitrogen. FITC conjugated goat anti-Armenian hamster, Abcam. DyLight™ 649 conjugated goat anti-rat IgG, BioLegend. Secondary antibodies were stained for 2 hours, washed 3 times, mounted with ProLong® Gold Antifade Reagent with DAPI (Invitrogen) and covered with a cover slip. Immunofluorescence staining was observed and photographed using a Zeiss 710 epi-fluorescence microscope. For quantification of lung infiltrating Gr1+CD79a+ myeloid cells, normal and metastasis bearing lung sections were stained with anti Gr1 (DyLight™ 649) and anti CD79-11 (FITC). Images of 20X magnification were analyzed. Gr1+CD79-11+ and Gr1+CD79-11^−^ myeloid cells were counted in normal lung, in uninvolved lung tissue from metastasis-bearing lung and in the lung metastases. Analysis was done for 3 samples in each group, assessing five fields (each 500 µm^2^) in each sample.

### Bone marrow cell differentiation

Bone marrow cells from naïve SCID mice were stimulated with anti-CD79a-V20 (5 µg/ml) for 96 h with or without added GM-CSF (20 ng/ml) in low (2%) normal mouse serum. At 96 h cells were harvested and stained for analysis by flow cytometry. Live cells were assessed by flow cytometry as the negative population for 7-AAD staining. The effect of anti CD79a-V-20 on myeloid cells differentiation status was assessed using immature (CD11b+Gr1+), granulocytic (Gr1+) and macrophage (F4/80) markers.

#### Statistical Analysis

Results were analyzed by ANOVA (post hoc test Dunnett's Multiple Comparison Analysis), or the Kruskal-Wallis test (post hoc test Dunn's Multiple Comparison Analysis) using Graph Pad Prism 5.0 b.

## Supporting Information

Figure S1 **Immature BM myeloid cells co-express the myeloid markers CD11b, Gr1 and the Gr1 subunit Ly6C.** A single cell suspension was prepared from naïve BM cells and analyzed by FACS for the different myeloid markers. The red box indicates the immature myeloid cells. All of the CD11b+Gr1+ cells are positive for Ly6C.
(TIF)

Figure S2 **CD79a is expressed on naïve immature myeloid cells.** (A) Cells isolated from the different organs of naïve Balb/c mice were analyzed by flow cytometry for co-expression of the myeloid marker Ly6C with CD79a/b as detected by the CD79-11 antibody. (B) Flow cytometry of immune cells in (A) showing that the CD79 subunit expressed on myeloid cells is CD79a (only detected by CD79-11 antibody) and not CD79b (detected by both CD79-12), whereas mature B cells express both markers. The box indicates the immature myeloid population. (C) Analysis of expression of additional B cell markers on leukocytes from bone marrow. Markers of mature B-cells are not expressed on the majority of the CD11b^+^ myeloid cells. The small population of CD11b^+^ cells that express B220 and CD19 probably represents plasmacytoid dendritic cells.
(TIF)

Table S1 **Cytokines secreted by the metastatic 4T1 and the non-metastatic 67NR cell lines.** Cell-conditioned media from 3 independent cultures of each of the 4T1 and 67NR cell lines (at 80% confluence) were collected and analyzed for cytokine level by quantitative multiplex cytokine array (Aushon SearchLight, MA). Cytokine levels are expressed in pg/ml and results are mean +/− SEM.
(TIF)
