# Supplementary figures and images for: Expression of the B-Cell Receptor Component CD79a on Immature Myeloid Cells Contributes to Their Tumor Promoting Effects
Source: PLoS One. 2013 Oct 16;8(10):e76115. doi: 10.1371/journal.pone.0076115 (PMC3797715; doi:10.1371/journal.pone.0076115)

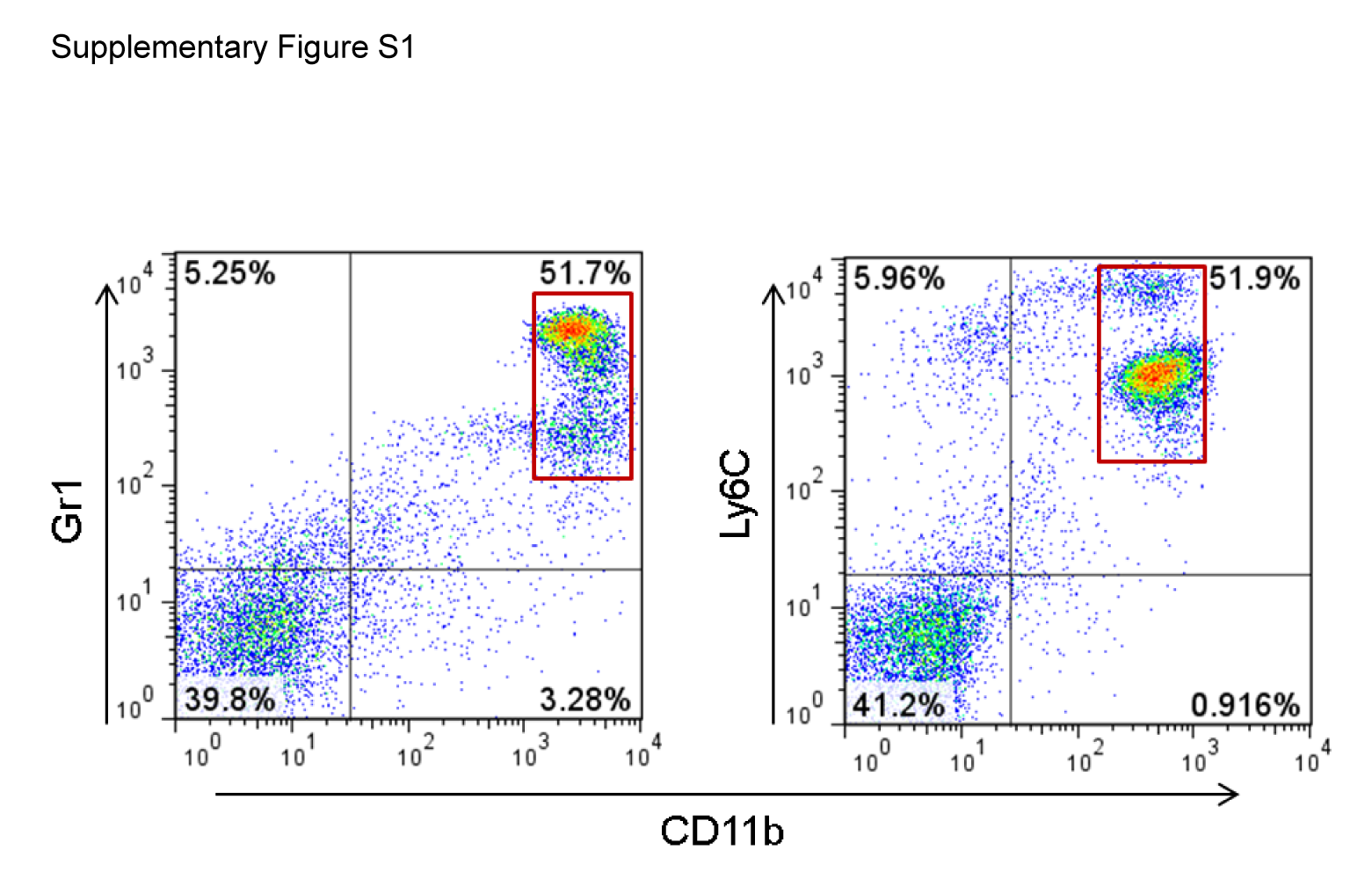

Supplement: Figure S1 — Immature BM myeloid cells co-express the myeloid markers CD11b, Gr1 and the Gr1 subunit Ly6C. A single cell suspension was prepared from naïve BM cells and analyzed by FACS for the different myeloid markers. The red box indicates the immature myeloid cells. All of the CD11b+Gr1+ cells are positive for Ly6C. (TIF) [file pone.0076115.s001.tif]

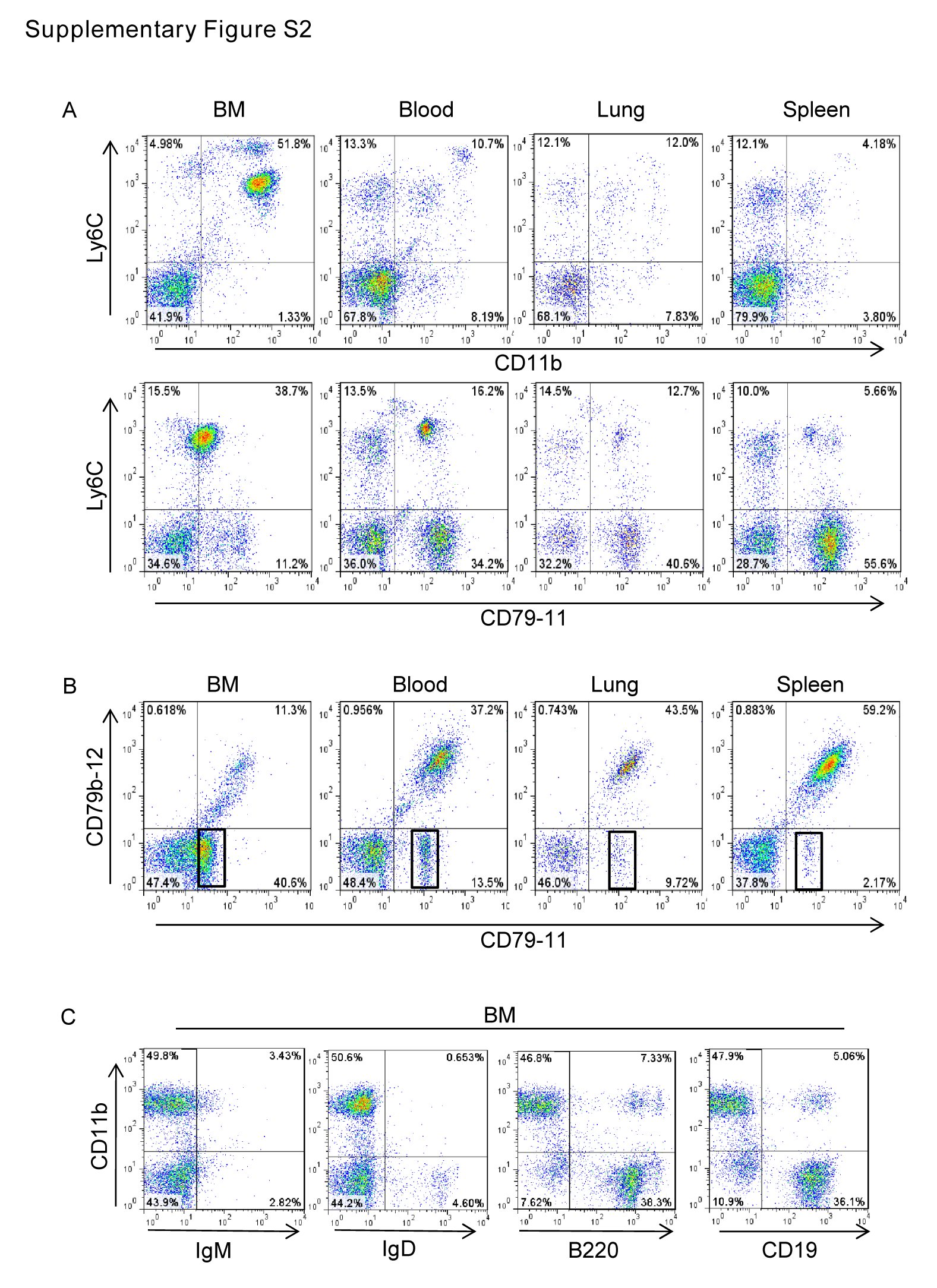

Supplement: Figure S2 — CD79a is expressed on naïve immature myeloid cells. (A) Cells isolated from the different organs of naïve Balb/c mice were analyzed by flow cytometry for co-expression of the myeloid marker Ly6C with CD79a/b as detected by the CD79-11 antibody. (B) Flow cytometry of immune cells in (A) showing that the CD79 subunit expressed on myeloid cells is CD79a (only detected by CD79-11 antibody) and not CD79b (detected by both CD79-12), whereas mature B cells express both markers. The box indicates the immature myeloid population. (C) Analysis of expression of additional B cell markers on leukocytes from bone marrow. Markers of mature B-cells are not expressed on the majority of the CD11b+ myeloid cells. The small population of CD11b+ cells that express B220 and CD19 probably represents plasmacytoid dendritic cells. (TIF) [file pone.0076115.s002.tif]

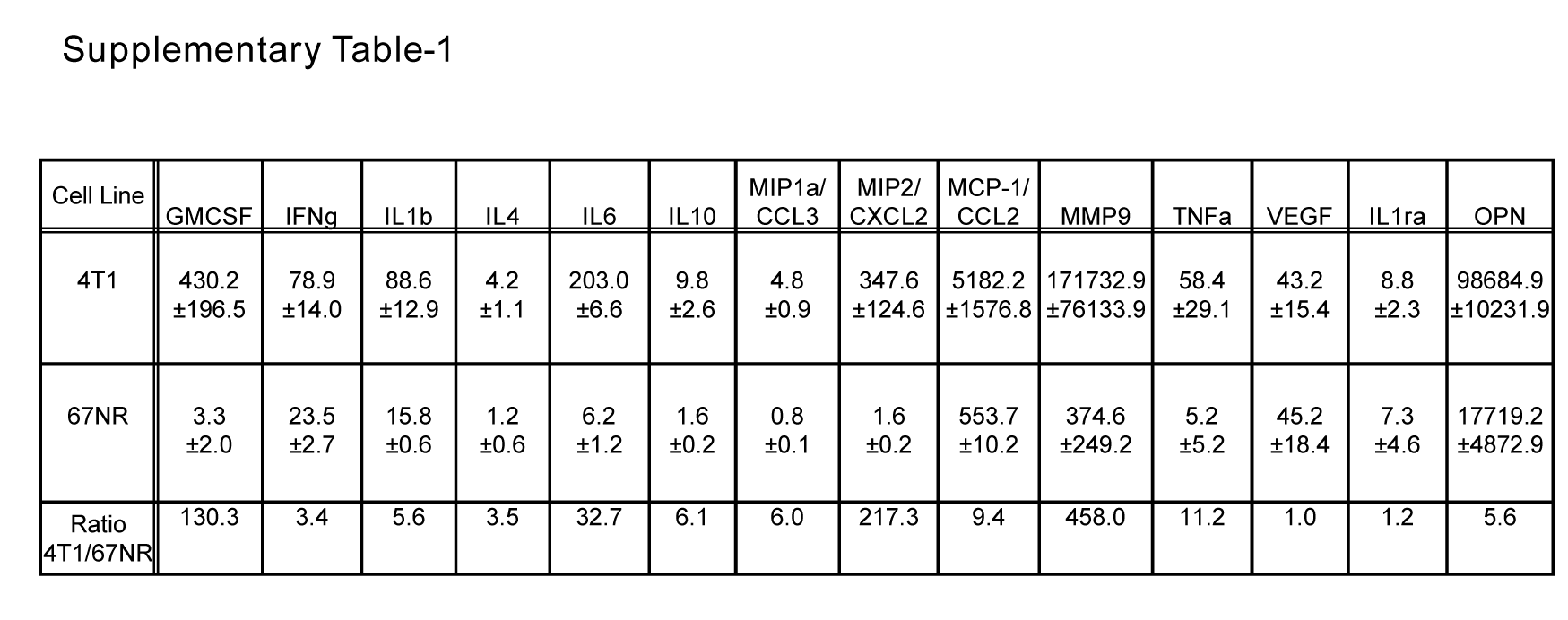

Supplement: Table S1 — Cytokines secreted by the metastatic 4T1 and the non-metastatic 67NR cell lines. Cell-conditioned media from 3 independent cultures of each of the 4T1 and 67NR cell lines (at 80% confluence) were collected and analyzed for cytokine level by quantitative multiplex cytokine array (Aushon SearchLight, MA). Cytokine levels are expressed in pg/ml and results are mean +/− SEM. (TIF) [file pone.0076115.s003.tif]
